# Beyond a neoliberal critique of hunger: a genealogy of food charity in Aotearoa New Zealand

**DOI:** 10.1007/s10460-023-10414-w

**Published:** 2023-03-15

**Authors:** Katharine S. E. Cresswell Riol, Sean Connelly

**Affiliations:** 1 Centre for Sustainability, University of Otago, Dunedin, New Zealand; 2 School of Geography, University of Otago, Dunedin, New Zealand

**Keywords:** Aotearoa New Zealand, Foodbanks, Food charity, Neoliberalism, Soup kitchens

## Abstract

Since the 1980s, foodbanks have become a widespread solution to addressing hunger within high-income countries. The primary reason for their establishment has been widely recognised as neoliberal policies, particularly those that led to massive cuts in social welfare assistance. Foodbanks and hunger have subsequently been framed within a neoliberal critique. However, we argue that critiques of foodbanks are not unique to neoliberalism but have deeper historical roots, meaning that the part neoliberal policies have played is not as clear-cut. In order to understand the normalisation of foodbanks within society, and gain a more extensive understanding of hunger and appreciation as to how this issue could be addressed, it is therefore important to gain a historical understanding of food charity development. In this article, we achieve this by presenting a genealogy of food charity within Aotearoa New Zealand, which witnessed a fluctuation in the use of soup kitchens during the nineteenth and twentieth centuries, and a rise of foodbanks in the 1980s and ‘90 s. Highlighting the historical parallels and major economic and cultural shifts that have allowed for the institutionalisation of foodbanks, we explore the patterns, parallels and differences exposed, and how they yield an alternative understanding of hunger. Using this analysis, we then discuss the wider implications of the historical foundations of food charity and hunger to better understand the role neoliberalism has played in the entrenchment of foodbanks, and advocate the importance of looking beyond a neoliberal critique in order to entertain alternative solutions to addressing food insecurity.

## Introduction

Over the last four decades, food charity in the form of foodbanks has become a widespread and permanent societal feature in the quest to address hunger within high-income countries, i.e., they have developed into the dominant food security response. Although the first formal foodbank is said to have appeared in the US in the late 1960s (Poppendieck 1996; Arizona Historical Society 2017), their substantial rise took place around two decades later. Research has revealed a common thread in their establishment: the rapid introduction of neoliberal social welfare modifications in the 1980s and ‘90 s devoid of features to mitigate the destitution that was to eventuate (Riches 1989, 1997a; Allen 1999; Poppendieck 2014; Cloke et al. 2016).

However, critiques of foodbanks based on societal blame and shame, the deserving and the undeserving poor, and the indignity of charity are not unique to neoliberalism but have deeper historical roots. Therefore, the enabling part that neoliberal policies have played is not as clear-cut. In this respect, it is important to gain a historic understanding of how food charity has developed, and the dynamics and multiple social and political factors involved, in order not only to comprehend how foodbanks have consequently become a “normal” feature of society but also to gain a broader understanding of hunger and how it might be addressed beyond what the neoliberal critique can provide.

In this article, we aim to demonstrate how the foundational approach and ideology towards hunger predates neoliberalism, and how, although neoliberalism has helped to solidify food charity and reduced resistance to this approach, it remains substantially unchanged. We intend to provide a different understanding to the current state of food charity and hunger, and explore what should be done to address food insecurity beyond the neoliberal critique. In order to achieve this, we will provide a genealogy of food charity within one high-income country, Aotearoa New Zealand (ANZ), highlighting the historical parallels and persistence of particular perspectives, and bringing into focus the major economic and cultural shifts that have allowed for the eventual growth and cementing of foodbanks into society.

To accomplish this, we will first consider how the literature frames foodbanks within a neoliberal critique. We will then present a genealogy of food charity in ANZ, within which we will consider the fluctuation in the use of soup kitchens during the nineteenth and twentieth centuries, and the rise of foodbanks in the 1980s and ‘90 s. This will be followed by a discussion of the patterns, parallels, and differences revealed between these periods and how they provide a different understanding of hunger. We then consider the wider implications of the historical foundations of food charity and hunger to better understand the role neoliberalism has played in the entrenchment of foodbanks, and how looking beyond a neoliberal critique could assist in sanctioning alternative solutions to addressing food insecurity.

## Neoliberal framing of foodbanks and hunger

In this section, we will consider how the critique of foodbanks is primarily framed from a neoliberal perspective, and how this contextualisation risks closing down alternative responses to and understandings of hunger. The four fundamental neoliberal framings we will consider are how foodbanks have (i) taken on state responsibilities and been engaged in social control on behalf of the state; (ii) concealed systemic issues related to hunger; (iii) contributed to a redefinition of the causes of hunger; and (iv) become the primary solution to hunger.

The neoliberal principles that enabled foodbanks to flourish included the value of market primacy, privatisation of state services, and self-reliance in the maintenance of welfare and wellbeing (Oak 2015). These principles justified government withdrawal from the social welfare sector, thereby enabling it to evade responsibilities in regard to social issues like hunger (Poppendieck 1996; Curtis 1997; Ghys 2018; Riches 2018; Lawson and Kearns 2020). Simultaneously, foodbank institutionalisation presented a façade of state competency and an effective social safety net (Wolch 1990), and gave the impression that public welfare—and neoliberal capitalism—were working. Foodbanks have thereby inadvertently not only permitted the government to shirk its obligations (Ghys 2018) but masked its failings (Curtis 1997; Riches 1999a).

Foodbanks have taken on the bureaucratic role of policing social welfare and subsequently been referred to as “contract providers of government mandated goods and services”, serving the state as opposed to those in need (Curtis 1997, p. 221), or legitimate extensions of the failing public welfare system (Riches 1999a; Riches 2018). It has even been argued that charitable interventions provide more flexible and efficient solutions than state-led social welfare (Weiss 2001). Riches (1986) has labelled them “agencies of social control” that meet the needs of capital, assisting in the disciplining of labour (p. 123). The introduction of eligibility requirements serve to control and constrain those who use the services provided (Wagner 1993), helping to cement foodbanks as a division of social welfare and, inadvertently, their services as a public entitlement, i.e., on par with government assistance.

It has also been recognised that the proliferation of foodbanks and their role in regulating hunger acts as a smokescreen, preventing the real systemic issues at hand from being addressed (Riches 1986). They act to conceal hunger, and their high-profile work has lulled people into believing that action is being taken (Caplan 2020). Denying the existence of hunger subsequently removes the need to name, define, or even care about this issue.

Simultaneously, foodbanks have rearranged, reconceptualised, and redesigned the meaning of hunger (Riches 1986; Hingtgen 1994; Caraher et al. 2014). There is a deep understanding within academia as to how foodbanks have depoliticised the concept (Curtis 1997; Riches 1997b, 2002; Power 1999), as explained by Riches (1997b): “[h]unger in western wealthy societies, it would seem, has effectively been depoliticized with profound personal, moral and social consequences” (p. 2). The arena for discussing hunger has shifted to the voluntary sector and is no longer addressed through public debate (Riches 1999b; Caraher et al. 2014).

Lastly, foodbanks have been identified as the primary gap filler between government support and outright hunger, and are a key component of the food system (Lambie-Mumford and Silvasti 2020; Silvasti 2015), as has been noted in numerous countries, e.g., the UK (May et al. 2019), the US (Duffy et al. 2006), and Australia (Booth et al. 2018). Today, there are foodbank associations, corporate sponsored conferences, and networks of private, public, business, and community actors, forming what Fisher (2017) labels the “hunger industrial complex”. At the same time, government funding is forthcoming, but it is clearly insufficient because of the continued presence, and even rise, of foodbanks. Food charity thereby not only continues to address the issue of food poverty but hides the fact that the government is failing to do so by giving the impression that this issue is being addressed.

### Foodbanks as the logical solution

Given the way that foodbanks have been aligned with neoliberal framings related to the role of the state and our response to hunger, it is inevitable that they have been positioned as the primary response to hunger and food insecurity. The diverse ways that they are socially embedded makes imaging alternative responses to hunger challenging: they crowd out any space for alternatives as they have been a resounding success as *the* solution to hunger, what Ronson and Caraher (2016) refer to as successful failures.

Foodbanks’ seemingly permanent integration into the social landscape has been attributed to the “socially acceptable” roles they play beyond simply food aid. Rather than acting simply as free food distributors, they are regarded as social service providers that serve as gateways to addressing the “real” issues behind the immediate need of hunger. As institutions on the front line of poverty, they are well placed to provide their clients with referrals to mental health, addiction, financial, and nutritional counselling, a role that has become increasingly institutionalised (Dave et al. 2016; Strickland and Whitman 2020).

Foodbanks have also further socially embedded themselves as a solution to food waste. They have strengthened their relationships with “food rescue” organisations that act as their food storage and distribution centres. Galli et al. (2019) describe how these relationships have integrated the social goals of ending hunger and preventing waste in parallel. Success is measured not only on the amount of food distributed but the amount of waste salvaged and/or carbon dioxide prevented from entering the atmosphere.

Finally, the foodbank has become an “important expression of community altruism” (Riches 1997a, p. 173). It has also been of benefit to business, as can be seen through the creation of food charity as “social enterprises”, e.g., the Trussell Trust (2020) in the UK, and the co-dependence between supermarket chains and foodbanks. Riches (2018) particularly targets the dependence of corporate food banking on industrial food waste, where supermarkets benefit from foodbanks solving their food waste problem in an economically efficient manner that also provides corporate social responsibility credit.

The way that foodbanks have been engaged as actors of social control on behalf of the state, their role in concealing systemic issues related to hunger, and their contribution to a redefinition of the cause and solutions to hunger illustrates how they are complicit in the rolling out and rolling back processes of neoliberal transformation (Peck and Ticknell 2002). Foodbanks are both a by-product of and fit within neoliberal policies (Cloke et al. 2016), and thereby embody a paradox: a solution to the negative social externalities of neoliberalism and enablers of the status quo.

Their alignment with the neoliberal agenda, and their ability to meet the needs of the state, business, and the public, have clearly played a major role in the institutionalisation and normalisation of foodbanks, and this has been recognised extensively within research. However, we argue that this only tells part of the story and that the roots of our dependence on the charity food model and underlying historical social and political factors play a significant role. Placing all the blame on neoliberalism obscures more fundamental questions about attitudes and approaches to hunger and how we allocate rights and responsibilities for food and hunger. In the next section we will examine the historical development of food charity within ANZ, unpack the culpability of neoliberal politics, and explore the additional social and political factors underlying food charity and hunger.

## Food charity genealogy

The following genealogy was compiled through archival research into food charity within ANZ primarily using archived newspapers as digitised copies, hardcopies and microfilm from the 1840s to 2020 through the Hocken library based at the University of Otago and via online databases. Relevant articles were searched for using the keywords “soup kitchen”, “soup kitchens”, “foodbank”, “foodbanks”, “hunger”, and “poverty”. These keywords were chosen based on the key concepts of the research and the dominant modes of food charity in ANZ. Other sources used included reports from the Church, NGOs, and the state, as referenced in the archival research.

We adopted a critical qualitative inquiry approach to select and guide the analysis of relevant articles (Denzin 2017). As such, our approach aims to not only locate and examine but also to challenge social inequalities and power relations based on the value orientation that social inequalities are systematic (Budd 2012). As this framework is value-laden, there is the risk of simply finding in the data what one wants to find, based on these values (Manning and Stage 2003). To address this risk, we were also guided by interpretive phenomenology that breaks data up into descriptive, linguistic, and conceptual components (Smith et al. 2012). The descriptive component accepts the content as is, taking into consideration events, descriptions, assumptions, and figures of speech. The linguistic component focuses attention on how the content and meaning are presented. The conceptual component incorporates interpretation of the overarching meaning that is being conveyed.

Relying heavily on newspapers to understand the historical role of food charity brought with it limitations, particularly in regard to the media’s propensity to selectively report on issues, and how the focus on foodbanks/poverty shifted across time. As a result, there were periods in time where there was relatively little media reporting, which does not necessarily mean that these issues had disappeared. In total, 217 articles and reports were included in the analysis.

What became apparent from analysis of the archival research were two periods in which food charity was particularly prominent: the 1860s to the 1930s and the 1980s to the present. In this respect, we have split the genealogy up into two main periods: the soup kitchen period, which ran from the 1860s until the 1930s, and the foodbank period, which ran from the 1980s until today. This latter period is then split up further into two phases: the 1980s to the 1990s, during which neoliberal policies were introduced, and the 2000s to the present in which they have been entrenched.

The period between the 1930s and 1980s is omitted because of the lack of articles including the keywords used. This apparent lull in the widespread need for food charity as reflected in the archival research is documented in literature and relevant reports. This period is referred to as ANZ’s long boom and it has a distinct geography (Conradson and Pawson 1997). It is characterised by a state of nearly full employment, economic expansion, a well-developed social welfare state, and Keynesian economics that subsidised economic activity in marginal places. The massive drop in those needing food charity post-Great Depression was attributed to the country’s economic upturn and implementation of the 1935 reforms of the Labour Party (Gosnell 1985). Societal problems, particularly due to high rent, did not disappear entirely, but, according to Christchurch City Mission’s 1946 Annual Report, the country’s advancements in social security had made an overwhelming difference to poverty in ANZ (Haworth 2019). Although, it should be noted that the social security system failed to support Māori to the same extent as Pākehā (McClure 1998).

### The legacy of colonisation

Just as it is not possible to consider domestic hunger without an understanding of rising inequality and poverty in ANZ, so it is not possible to consider poverty and inequality within ANZ without acknowledging colonisation and its continued impacts (Moewaka Barnes and McCreanor 2019). ANZ’s legacy has origins that include the violent alienation of the indigenous people from their land and resources, leading to a loss in their spiritual, cultural, and economic bases (Cram 2011). An in-depth analysis is beyond the scope of the article, however it is important to recognise that the capitalist economy introduced by the British was founded upon unequal power relations and accumulation at any cost. Māori were forced to move from a system based on sustenance, reciprocity, and community to one based on scarcity, individualism, and private property (Poata-Smith 2019). Their food sovereignty was destroyed through the loss and destruction of land, prevention of access to traditional foods (Barnes et al. 2018), and enforced nutrition transition, brought about by a complete disregard for Māori culture and disrespect for their basic rights (Petrie 2006).

Colonisation continues to have deleterious impacts on Māori to this day, and remains a pivotal reason as to their comparative disadvantage. The legacy of colonialism has been the “differential distribution of social, political, environmental and economic resources and well-being within this country with Māori bearing the brunt of disparities in many areas” (Cram 2011, p. 156), e.g., political disenfranchisement, land misappropriation, population decline, and socio-economic marginalisation (Walker 1990). Similarly, the injurious impacts of colonisation on Māori food sovereignty resonate today: access to traditional foods remains limited (Bowers et al. 2009; McKerchar et al. 2015); the detrimental impacts of the nutrition transition are ongoing (see, for example, Bell 2017; Best Practice Advocacy Centre 2018); tensions exist between Māori and state models of resource management, especially in regard to customary fisheries resource management tools and proprietary water rights (Pehi et al. 2009; Waitangi Tribunal 2019); and land ownership disputes prevail (Mutu 2018).

### Soup kitchen period: 1860s–1930s

Sold as the land of “milk and honey”, ANZ was promoted as an escape from the starvation and inequality that ravaged Britain in the nineteenth century (Campbell 2020). However, for the settlers who arrived in Port Nicholson in 1840, there was not enough paid work or food (Phillips and Hearn 2008). Dependent as most were on wage labour, destitution was high from the outset, and only increased throughout the mid-1900s on account of trade depression, a rising population, and a lack of social assistance (Sutch 1941).

A soup kitchen system was proposed in the late 1850s in response to a petition concerning the country’s lack of employment, but was expeditiously repudiated by the local council as unacceptable compensation to paid work (Provincial council 1859). This was endorsed by the country’s strong colonial credo of personal responsibility and egalitarianism: success was considered possible for all because land was available to all (at the expense of dispossession of Māori); individual moral failings were to blame for poverty (Tennant 2018). A policy-based response to sanction poor relief—the Destitute Persons Relief Ordinance of 1846, followed by the 1877 Destitute Persons Act—subsequently solidified this stance by making households liable for their indigent relatives.

With a national response lacking, soup kitchens emerged in the mid-1860s as the primary channel of poor relief (Untitled 1867). Victim-blaming prevailed based on the reasoning that those using soup kitchens were capable of working (Flax hackle benevolent society committee 1867). Considered contrary to a system based on rights and having failed in their “humane object” (Destitution 1866, p. 4), they symbolised a level of abject destitution (Prosperity of Auckland 1865), and, under the dichotomy of un/deservingness, stricter measures were enforced to ward off “imposition and idleness” (Papakura flats 1867, p. 5).

A departure from this stance appeared in 1867 when Auckland introduced the Fund for the Relief of the Sick and Destitute to support the running of soup kitchens (Flax hackle benevolent society committee 1867), followed by the 1868 Sick and Destitute Act, which imposed a general tax for soup kitchens. This radical move from voluntary donation to enforced taxation was not welcomed across the board: classified as a waste of money, it was reasoned that obligatory giving “destroyed the virtue of charity” (Relief of the sick and destitute 1868, p. 4), and, if the issue of assisting the sick and destitute could not be dealt with in a charitable manner, “it was their duty to look upon it as one of the general subjects of government” (Important meeting at Otahuhu political aspect of the province discussed 1868, p. 4).

Soup kitchens were accused of having a “deleterious influence”, leading to dependence and a loss of self-respect (Almsgiving 1872, p. 1), even when the working poor became part of the soup kitchen clientele (Uttley 1997). Population growth in the 1870s, coupled with the ongoing recession, gave rise to social disparities becoming increasingly entrenched (The sweating system 1888), exposing the egalitarian mantra as seriously flawed, and further highlighting the need for state assistance (Oliver and Williams 1981).

The first major economic depression of the 1880s, subsequently known as “the regime of the soup kitchen” (Address at Waimate 1913, p. 10), was a decade of deplorable poverty, defined by dire working conditions and paltry wages for those in employment, and soup kitchens for those not (Hayward and Shaw 2016). Public efforts were made to publicise the systemic nature of hunger in terms of government mismanagement, and concerns were raised that destitution would worsen if charity supplanted state responsibilities (Christchurch Benevolent Association 1880; Soup kitchens, & c. 1880). Opposition to soup kitchens also came from the users themselves. In response to a soup kitchen opening in Christchurch, the Committee of the Canterbury Unemployed publicly voiced their grievances, labelling it “the thin end of the wedge of pauperism”, and accusing it of exacerbating not diminishing poverty (Meeting of unemployed 1880, p. 3). Their call was for “employment—no matter at what wage” (“The unemployed” 1880, p. 6).

Increased state involvement materialised in the mid-1880s with the subsidisation of the newly established district Hospital and Charitable Aid Boards, systematised throughout the country via the Hospitals and Charitable Aid Institutions Act of 1885. However, a distinction between the deserving and undeserving poor was made through provision of indoor relief for the “helpless” and elderly poor, and outdoor relief for the unemployed, deserted wives, and widows (McClure 1998). In addition, adequate assistance was not guaranteed as funding was dependent on local governing board funds and the disposition of the relieving officers (Oliver and Williams 1981; McClure 1998). There was such apprehension over not replicating the shame of the English Poor Laws that the continuation of the milk and honey façade was deemed more important than preventing starvation (for example, “Auckland” 1879).

Although the Liberal era of the 1890s brought with it economic recovery, and despite ANZ possessing the world’s highest standard of living by the turn of the century (McClure 1998), urbanisation between 1880 and 1900 led to a rise in poverty and hunger. However, by the end of the nineteenth century, only the elderly were being granted official state support through the 1898 Old-age Pensions Act; others facing desperate hardship were left to contend with Charitable Aid Board assistance, which continued to be granted stringently due to a lack of trust in those asking for help, and concern that too much assistance would lead to it being regarded as an entitlement on par with the old-age pension and government assistance (Tennant 1981).

Countrywide pockets of indigence drove Church leaders to address hunger with the re-establishment of soup kitchens for the unemployed and food distribution for the destitute (Oliver and Williams 1981), and, as the twentieth century dawned, they increased throughout the country (Local and general news 1905; The unemployed” 1908; The unemployed 1909). Although some felt that such charity was even below Christian obligations—“The primary duty of a Christian Church was not to descend to the level of a soup kitchen, but to reach the man and enable him to help himself through Christ” (Cole 1909, p. 3)—the Church continued to take an active role. An article published in 1905 by the New Zealand Times entitled “Feeding the Hungry” focused on a soup kitchen run by Mother Mary Joseph Aubert. For the past three years, it had been serving on average 80 to 90 people daily. She had a refreshing take on those who were inebriated, i.e., the undeserving—“What then? That is when they need the soup most!”—as well as on “cheats”: “There are ‘loafers,’ no doubt, but if there be hungry men in a community, ‘twere better to be cheated by a hundred loafers than that there be no heart open for a poverty-beaten wretch” (Feeding the hungry 1905, p. 7).

As well as soup kitchens, there are early signs of foodbank style operations being implemented by the Church: Wellington City Mission, which opened its doors in 1904, handed out “Christ Cheer food parcels” (Gosnell 1985). More formal monitoring was put in place, for example, in 1908 the Salvation Army opened a soup kitchen in Ōtautahi-Christchurch at which “as proof of bona fides, [recipients] must leave their names and addresses for future reference” (A soup kitchen 1908, p. 5). There was also continued pressure to acknowledge poverty as a societal problem, for example, by the Presbyterian Church (Dougherty and Thomson 2006), but this was disputed with the argument that, if the state were to take on more responsibility, the result would be “a nation of paupers” (The H. B. tribune: The medical congress 1911, p. 4), as well as claims that poverty was being exaggerated and therefore the presence of soup kitchens was a humiliation (No demand for soup 1908), on par with “the methods of the English slums” (An army officer’s testimony 1909, p. 3).

However, apart from the outbreak of influenza in 1918 leading to a discernible hike in usage (Influenza epidemic” 1918; Soup kitchens” 1918; Voluntary help” 1918), there followed an apparent lull in those seeking charitable food assistance. For instance, it is reported that Wellington City Mission, which had started up in the early 1900s, served meals mainly for the elderly, with only the occasional impoverished child, unemployed individual, or widow requiring assistance (Gosnell 1985).

The situation changed in the 1920s with the onset of the Great Depression: this was a time of increased unemployment, paltry incomes, and a loss of homes, domestic security, and spending power (Gosnell 1985; Haworth 2019). As hospital boards and voluntary groups quickly became overstretched with the amount of demand (McClure 1998), the need for soup kitchens re-emerged nationwide, and as more organised affairs, with citywide card indexes to prevent people from using multiple soup kitchens and public food appeals (Gosnell 1985; Pollock 2013; Haworth 2019).

The Unemployment Committee was particularly vocal as to how degrading and improper soup kitchens were in place of paid labour (Unemployment committees 1930). In 1932, riots even took place in response to the high levels of unemployment and lack of adequate support (Dunedin food riot 1932). However, the official reaction of central government to these desperate acts sought to silence rather than support the aggrieved: the Public Safety Conservation Act allowed for the suppression of democratic norms if necessary, and the deportation of able-bodied unemployed male workers to work-for-the-dole labour camps located in the countryside (Trotter 2014).

Soup kitchens essentially disappeared from the archival research, which may reflect improved economic prosperity, but could also reflect a shift in media attention at the time. Wellington’s Compassion Soup Kitchen, run by the Sisters of Compassion, appears to be the only standardised operation remaining by the late 1930s. Although undoubtedly, of course, smaller, less formal soup kitchens and community meals also remained. During the 1950s and up until the ‘80 s, food chits were only given out occasionally, and those requiring free meals were, again, primarily the elderly (Gosnell 1985).

In summary, throughout this period, mass emigration into the country and the lack of employment led to widespread impecuniousness. Yet, state assistance was not forthcoming, justified by capitalist, colonial tenets that claimed that, in this land of equal opportunities, it was a deficiency of the individual not the state. Even arguments against charity focused on dependency, placing the onus on the individual and their apparent propensity to freeload if given the opportunity.

Suggestions of establishing soup kitchens in lieu of social assistance were met with public defiance, particularly as charity, symbolic of social stratification, was at odds with ANZ’s claim of egalitarianism. Yet, during the economic depressions of the 1880s and 1920s, soup kitchens were an important part of welfare assistance, even though those who disapproved most fervently were the unemployed who were expected to use them. We now jump forward in time to the 1980s and the resurgence of the need for agencies outside the state to provide food for the hungry.

### Foodbank period: phase one—neoliberal initiation (1980s–1990s)

Phase one of the foodbank period possesses certain parallels with the preceding period, as will be discussed below: with shortcomings in state assistance, charitable aid was deemed necessary, but it remained distinctly unwelcomed. A major point of difference, however, was that the point of resistance changed from those who were hungry to those attempting to help those who were hungry.

The neoliberal reforms introduced in the late 1980s and 1990s led to the need for food charity returning with such force that it was compared to that of the pioneering days (Mahony 1990). The “especially doctrinaire” adjustments (Craig and Porter 2006, p. 222) consisted of market liberalisation, free trade, restricted government, narrow monetarist policy, a deregulated labour market, and fiscal restraint (Kelsey 1996). Although newspapers reported on the need for soup kitchens being stronger than ever (Give us this day our daily bread 1989), foodbanks were to emerge as the “public symbol of charity” (Kelsey 1996, p. 292).

The first foodbank materialised—unintentionally—in 1983 in Auckland. Established at the city’s railway station over the Easter weekend, parishioner Janet Bromley had regarded it as a “one-off” action to assist people over the winter, but, as she explained, “once I’d turned the tap on, it wouldn’t turn off” (Randerson 2015, p. 6). Between 1989 and 1992, the number of foodbanks throughout the country rose rapidly (Mackay 1994; Social Policy Agency 1994); by 1991/2, foodbanks emerged as independent organisations that dealt only with food parcels and employed staff for this specific purpose (Dougherty and Thomson 2006; Foodbank staff busier than ever 1998).

In response, the government’s Social Welfare Minister at the time was not only quick to charge foodbanks with generating their own demand but also those obtaining such assistance as not truly requiring it (Ansley 1992); blame was placed on money mismanagement (Richardson 1992), despite nationwide research proving otherwise (Hubbard 1991; More using food banks, survey finds 1991). Simultaneously, the rise in charity assistance was welcomed as it enabled the creation of a more caring community (Reid 1992). The foodbank was also in line with the neoliberal ideal of self-reliance, but in the capacity that individuals should become dependent on their immediate families and voluntary organisations (Kelsey 1995).

Nevertheless, in February 1992, the government-initiated People’s Select Committee was established to investigate the effects of the recent policy changes (Craig et al. 1992). In its final report, “Neither Freedom nor Choice”, the committee observed that, if foodbanks continued to take on state responsibilities, “[n]ot only would charity become further entrenched into the existing welfare system, and present policies legitimated, but the access people have to basic human need would depend solely on the decisions of others” (p. 22). Subsequent research conducted by the Social Policy Agency (1994) supported this stance, concluding that it was likely that foodbanks would “become entrenched as a more enduring component of the welfare system unless action [was] taken to reduce demand for their services by some form of public provision” (par. 72).

Despite these cautionary reports, that same year foodbanks had become a “growing industry”, with over 300 organisations involved in collecting and distributing food (MacDonald 1994, p. 5). This was in part due to the sheer number of people requiring assistance (e.g., Food bank need ‘entrenched’ 1996; Chaotic times at foodbank 1997; Slade 1997; Rise in food bank use shows poor ‘plight’” 1998; Foodbank ‘sign of the times’- Co-ordinator 1999). As need increased, so did the sense that foodbanks were not only a viable alternative to social welfare (Food-bank use in Christchurch rising 1996) but a branch of it: by at least 1998, foodbanks were being referred to as “social services” (Calcott 1998, p. 8) and “welfare agencies” (Welfare agencies brace for busy period 1998, p. 6), and the government was no longer seen as the final port of call because this role had been adopted by foodbanks (Food grant pact an outrage, says MP 1997).

The perception of foodbanks playing the role of welfare agencies was reinforced by New Zealand Income Support Service (NZISS)^1^ directly referring individuals to foodbanks when they were no longer prepared to assist (Big rise in number using food bank 1995; Food bank under pressure 1997; “Food bank usage ‘shows desperation’” 1998; Food banks to close in protest move 1996). In light of the fact that beneficiaries were being consistently sent to foodbanks without their social welfare entitlements being fully met by the NZISS, a 1994 report entitled “Passing the Buck” was commissioned by Wellington’s Downtown Ministry, which warned that a “formal relationship” between government and foodbanks should be discouraged (Barwick 1994, p. 33). Even so, that same year, the Minister for Social Welfare pushed for the strengthening of liaisons between foodbanks and the NZISS (MacDonald 1994).

Those running foodbanks, primarily churches, not only added to their social entrenchment but perpetuated a victim-blaming mentality. Both were apparent in their social service provider role, which was reinforced through the provision of additional services, particularly in the form of budgeting and self-help advice (Down and out in Dunedin 1991) under the assumption that people were hungry due to personal choice or a lack of skills. An initial example was Christchurch City Mission’s 4C advocacy programme (Gee 1999): carried out in alliance with the government, its aim was to “wean beneficiaries off a dependency on foodbank parcels” (McCarthy 2003, p. 4). In a paradoxical move to “reduce the shame-and-blame attitude”, participants were informed that they were in this predicament due to “entrenched habits and lifestyles” (McCarthy 2003, p. 4).

Entrenchment of blame and the view of the deserving/undeserving poor was also achieved through foodbanks’ increased bureaucratisation, including strict registration processes that involved the provision of personal details and reasons for assistance (Petrovic 1997; Calcott 1999). As with the response to the rise in soup kitchens, there was increased communication between foodbanks within cities in an attempt to prevent individuals collecting food from multiple locations (Ansley 1992; Dougherty and Thomson 2006). Such measures bolstered the negative stereotype of the foodbank *ab*user and unfavourable assumptions around who used foodbanks and why.

However, the 1990s were also a time of politically fuelled action in recognition of the bind those running foodbanks recognised they were in. The New Zealand Council of Christian Social Services (NZCCSS) explained how.

Christian agencies face a moral dilemma in times of social crisis. It can be argued that we are letting the government off the hook by stepping in to fill the charity gap. We feel obliged to respond to immediate needs of the community. We must speak out for those people whose humanity is being violated. (New Zealand Council of Christian Social Services 1992, p. 4)

Ruth Smithies, from the Catholic Office for Justice, Peace and Development, Whanganui-a-Tara-Wellington Archdiocese, explained that the Church was not attempting to play politics (“Church worker berates MP” 1995, p. 20). However, she recognised that its work must go hand-in-hand with the establishment of just structures so that it was not depended upon. On the other hand, concerns that foodbanks were stopping radical action were raised: Russell Toyne of Manurena’s St. Vincent de Paul commented on how “[g]iving alms can be a cheap form of charity” (Christchurch among most needy for Salvation Army parcels 1995, p. 2).

As discussed by historian Peter Lineham, during the 1990s, Christian organisations were particularly vocal: the NZCCSS has even been hailed as a “more effective pressure group on social policy than any non-government organisation” during this time (Lineham 2004, p. 147). Before the 1990 election, the NZCCSS met with opposition party members and was assured that changes within economic and social policy would proceed more slowly than in the ‘80 s. However, when this did not transpire, Church leaders created their Social Justice Statement (Davis et al. 1993). Released in advance of the 1993 General Election, it requested that the state review benefit rates and the tax structure, and that priority be given to those in the lower socio-economic bracket. The document “immediately threw up controversy” and was attacked for being left-wing, communist propaganda by staunch defenders of the neoliberal restructuring (Lineham 2004, p. 163).

The following year, a Foodbank Co-ordinators Conference was held in Auckland (Food banks: part of the problem 1994). No longer prepared to assist on the current scale, the 180 delegates present agreed that they would change the emphasis of their work from simply meeting food parcel demands to changing the overall system. The Unemployed Workers’ Rights group suggested that foodbanks should close, but, although delegates agreed with the overall sentiment, it was generally felt that this would lead to too much additional hardship.

However, two years later, action was taken in line with this proposal. Reports that just under 20 percent of New Zealanders were living below the poverty line (Stephens and Waldegrave 1995) prompted foodbanks to call for a nationwide “strike” as part of National Action on Poverty Week 1996, during which they would refuse to collect or distribute food (Christchurch food bank to join week-long strike 1996; Rally focuses attention on need for food banks 1996). Instead, people would be directed to the Department of Social Welfare where individuals would have to be given a food grant as the closure of the foodbanks should be considered an “exceptional circumstance” (Christchurch food bank to join week-long strike 1996, p. 4). However, many foodbanks decided not to participate, concerned that only their clientele would suffer (Christchurch food bank to join week-long strike 1996; Rally focuses attention on need for food banks 1996). This included four foodbanks in Dunedin, where a rally was held in the city centre’s Octagon instead: as well as speeches, a cache of 1200 balloons was popped, each representing a food parcel that had been given out that year in the city, feeding approximately 36,000 people (Rally focuses attention on need for food banks 1996).

In summary, during this foodbank phase, the entrenchment and bureaucratisation of foodbanks was characterised by the widespread adoption of managerialism where those seeking basic rights were treated as customers. There was also politically founded resistance, primarily led by the Church, in contrast to the previous period where resistance was led by soup kitchen users, but they were also complicit in the processes of managerialism and bureaucratisation, adopting the client-focused rationale and seeing themselves as service providers. This reflects the shift in focus from the existence of poverty and hunger towards the institution of charity. Although the political action failed to prevent foodbank entrenchment, it was still present, in contrast with the following phase.

### Food bank period: phase two—corporate appropriation (2000s-present)

What is particularly apparent in this second phase is foodbank corporate appropriation and foodbanks’ own transformation into business-like entities. As the twenty-first century approached, foodbanks had become even more established and were recognised as “New Zealand’s largest growth industry” (Hartevelt 2009, p. 1). However, there remained unease around the fact that a “temporary charitable response had become a fixture” (McCrone 2008, p. D3), particularly by those running the foodbanks.

Foodbank use continued to rise (e.g., Clarkson 2000; Mission foodbank use rises sharply 2002; Food bank use still rising, despite apparent upturn 2009; Schoultz 2015; Feinberg 2018), with a noticeable peak during the 2008 Financial Crisis; while the subsequent recession was over by mid-2009, the Salvation Army saw an one hundred percent increase in the need for its services to 2012 (Franks 2020). This rise was accompanied by individual blame around foodbank users’ inability to budget or cook. At the third Otago Hidden Hunger Forum held in 2000, attempts were made to address such food poverty myths (Myths targeted 2000). However, the view that the crux of the problem was money-mismanagement persisted (McCurdy 2001).

Entrenchment of foodbanks deepened through stronger partnerships with government services (New Zealand Council of Christian Social Services 2008). In 2002, the Ministry of Social Development strengthened relationships in a move to *rid* the country of foodbanks in the form of a 3–5 year Foodbank Strategy that institutionalised funding and management. A Foodbank Social Coalition Fund was subsequently established through the 2002 budget, and Work and Income New Zealand (WINZ)—previously NZISS—became more active in managing referrals and working with foodbank providers to reduce dependence on their service through the presence of WINZ case managers in foodbanks and the funding of additional programmes. According to a 2008 NZCCSS report, the strategy had “no significant impact on reducing the need for foodbanks” (New Zealand Council of Christian Social Services 2008, p. 5).

Foodbanks’ social service role also became more pronounced, and there was a shift from the need for food parcels being a problem to using food parcels effectively (Spratt 2004; Davis 2006). In addition, foodbanks underwent significant transformations, an observable way being through their rescaling. A particularly prominent example is 0800 HUNGRY: established in Christchurch in 2001, this “dial-a-food-package” foodbank (Key to address food charity: 0800 HUNGRY 2007) claims to be “New Zealand’s largest foodbank” (0800 HUNGRY 2002), with aspirations to expand into a “mega-foodbank cum community centre” (The $12 m Christmas wish 2015, p. C8). Foodbanks also became less conspicuous: established in 2012, Feed the Need introduced its Pātaka—the *te reo* Māori word for pantry—Programme within schools (Feed the Need 2021). This not only assisted in normalising need further but, by positioning food charity within schools, it increased its socially acceptability and untouchability.

Foodbanks’ relationship with businesses also transformed and strengthened. Whereas in the late 1990s foodbanks were primarily funded by and obtained donations from the churches that ran them as well as individual contributions (Uttley 1997), by the twenty-first century, supermarkets played a more prominent role. Donating transformed from physical food donation bins in stores in the mid-90 s (Farrimond and Leland 2006) to a more convenient online option (Shaw 2019). Supermarkets then became even more adept through their excess “waste”. Although it appears that surplus food was being used for food charity as early as the mid-90 s (Big increase in requests for food parcels 1995), it was not until the late 2000s that its use was institutionalised with the emergence of “food rescue” operations. These operations promote themselves as solving two problems: food waste and food insecurity (Food rescue: solving two problems 2018). In 2020, they became “NZ Food Waste Champions” in line with Sustainable Development Goal 12.3, for which “feeding people” is included as a “win” of addressing food waste (NZ Waste Champions 12.3 2020).

Foodbanks also partnered up with business, particularly for “foodraising” campaigns. An early example was the Village-Hoyts-Wattie’s Cans Film Festival, which ran annually for 22 years and enabled the public to pay for a cinema ticket with a can of food (van Beynen 2016). However, during the 2010s, there was a significant boost in other business participation as foodbanks were associated with good corporate citizenship (Sallies, Smiths City join forces 2010; Chickens and pavs cheer struggling families 2011; Donating for good 2012; Cans of food a ‘massive’ help” 2015; DINEAID 2020).

A more alarming case, however, can be found in attempts to replace the government’s food grant with a standardised food bag (Beneficiaries being delivered My Food Bag meals as part of Government trial 2019). Supplied by the business “My Food Bag”, a trial of one thousand kits, voluntarily received, was conducted in early 2020 in Auckland. Not only was it recognised as another way to avoid giving money to those in need (Edmunds 2019a), but it was found that the food box minimised human contact, did not cater to specific dietary requirements, and lacked non-food essentials, e.g., toiletries and washing products (Beneficiaries being delivered My Food Bag meals as part of Government trial 2019). Although unsettling, such private enterprise could be considered a logical evolution, despite the fact that it was more expensive to procure food through My Food Bag than directly from a supermarket (Edmunds 2019b).

The 2010s also saw the introduction of more prominent community events. For instance, Dunedin’s first annual Octacan was established in the winter of 2010, for which the public were encouraged to bring cans to the Octagon in the city centre (“Foodbank appeal with a ‘can-do’ attitude” 2010). This was followed by “canstruction” in 2013 in Christchurch, a yearly-held competition in which teams compete to build a sculpture entirely out of cans (If anybody can, a Crusader can… 2013). In addition, foodbanks instigated their own foodraising events in the form of annual foodbank drives (Rudd 2007; Taieri Food Bank drive successful 2007). All of these initiatives served to reinforce the position of foodbanks as the logical solution to hunger and acceptance of their role.

Compared to the events described above, political activity became less evident as publications seemingly replaced protests, including a report commissioned by the Child Poverty Action Group entitled, “Hard to Swallow: Food-bank use in New Zealand”, which advocated the need for school breakfasts in low decile schools. However, Presbyterian Support Otago’s (2002) report, “How Much is Enough? Life below the Poverty Line in Dunedin”, which included stories of those dependent on foodbanks in the city, prompted Dunedin City Council to sponsor a forum on poverty. This led to the development of PANDO: Poverty Action Network Dunedin-Ōtepoti (Dougherty and Thomson 2006), a network of 35 organisations working together to eliminate poverty in the city. Poverty even became an election issue, leading to a 2004 survey of people who lived in low-income private rental housing in the city and the subsequent report, “Old, Cold, and Costly?” (Povey and Harris 2005).

The collective voice of the Church remained emphatic about the need for political action on hunger and poverty, and today consists of four main actors: the NZCCSS, arguably still the most prominent advocate; the National Church Leaders Group, which meets regularly with the government; the City Mission; and the Salvation Army, which started its yearly and influential “State of the Nation” report in 2008. More recently, the Kore Hiakai Zero Hunger Collective was launched in 2020. Under the tagline, “for an Aotearoa where everyone has dignified access to enough good food”, it aims to address the root causes of food insecurity using a collaborative approach (Kore Hiakai 2020). However, in response to the COVID-19 pandemic, it joined forces with the government in 2020 to assist with the provision of charitable food relief (Ministry of Social Development 2021).

At a governmental level, Jacinda Ardern’s message has been one of compassion and kindness. However, not everyone has benefitted from this message (Khalil 2020). The widening gap between the rich and poor remains a particularly jarring issue in ANZ. A 2017 report published by the Victoria University of Wellington provides a graph of Gini coefficient measurements spanning from the 1930s to 2014: notably, the lower level of the late 1930s has never been re-attained, and, apart from a spike in 1999/2000, it has remained relatively constant from 1994 after increasing during the late 1980s (Creedy et al. 2017).

The state has made its own moves to “humanise” poverty, rebranding the concept into a more socially acceptable form: child poverty and, through association, that of families. Ardern not only made child poverty a major focal point of her party, but assigned herself as Minister for Child Poverty Reduction (Roy 2017). However, by association, the poor adult remains demonised: the palatability of such poverty can be found in the blamelessness of the child; (unemployed) adult hunger cannot exist as child hunger exists because the former is regarded as self-inflicted.

The state has also tried to humanise the economy: in its 2018 Budget Policy Statement, Labour introduced the Wellbeing Budget (New Zealand Treasury 2018). In an attempt to look beyond the soulless disposition of the gross domestic product, it has been sold as an “intergenerational approach that seeks to maintain and improve New Zealanders’ living standards over the long-term” (New Zealand Treasury 2018, p. 1); economic growth alone will no longer be of paramount importance. However, there is an obvious paradox: well-being is being advocated within an economic system that actively debases well-being. If neoliberal policies have generated most of the negative social outcomes, it stands to reason that it will be hard if at all possible to attempt to fix them within the neoliberal realm.

Another glaringly obvious point of contention is the welfare system: moves may have been made towards a well-being budget, yet no truly impactful advances have emerged to create a welfare system based on well-being let alone human dignity. In 2018, the Welfare Expert Advisory Group was established by the Ministry of Social Development to assist in the creation of a welfare system that ensures an adequate income and standard of living. The final report,“Whakamana Tāngata—Restoring Dignity to Social Security in New Zealand”, concluded that, to create a fair system that safeguards and instills dignity, there needed to be a complete overhaul of the current structure (Welfare Advisory Group 2019). The response by the Labour Government to this more than 200-page document has been minimal, and described as disappointing (Davison 2019), dismal (Bradford 2019), and pathetic (Robson 2019).

To summarise, although this sixth Labour Government has made moves to reignite the state’s more compassionate characteristics in the form of the Wellbeing Budget and its focus on child poverty, fairness, and neoliberalism are incompatible, and they remain a neoliberal government. Over the last 20 years, food security issues remain closely tied to food charity. In this time, food charities, businesses, and individual donors have gained prominence and play a greater role in debates and action about hunger, while the foodbank user has been further hidden behind additional social justice causes.

## Patterns, parallels, and differences

We will start this section by discussing the significance of breaking down the history of food charity in ANZ into these three specific sections in respect to formulating a more comprehensive understanding of food charity’s positioning in society today. What is apparent during the soup kitchen period is how these charitable institutions ebbed and flowed with economic crises in the 1870s to ‘80 s and 1920s to ‘30 s. Attempts to introduce soup kitchens—even when hunger abounded—were met with widespread contempt and actively resisted because they were a visible shame that all society felt. Simultaneously, food charity was seen as a temporary necessity tied to specific economic downturns.

Foodbanks, on the other hand, mushroomed with the introduction of neoliberal economic policies in the 1980s and ‘90 s. What is particularly striking in the foodbank phase is the normalisation of food charity as it became more indicative of community and capital. Foodbanks were unabashedly institutionalised, celebrated, and appropriated by businesses, increasingly included within corporate agendas as well as transforming into business-like entities themselves. Neoliberalism specifically led to foodbanks being subjugated by market necessity, enabling hunger to be capitalised on.

These periods bring to the fore how certain attitudes towards food charity have continued despite wider economic, political, and social change, while others have been amplified or transformed, revealing the positioning of food charity beyond neoliberalism. In order to demonstrate the value of this in understanding the current state of foodbanks and hunger, we will reflect on three main points of comparison: (i) individualistic narratives; (ii) points of resistance and political action; and (iii) individualisation of blame.

### Individualistic narratives

Efforts to appear a prosperous colony have been detrimental to those benefiting least from any abundance on offer. Political inaction led to widespread starvation in the nineteenth century, firstly, through the lack of social welfare provision, buoyed by fear of reproducing England’s Poor Laws, and secondly, through the demonisation of charity due to its links to British class ideals. In comparison, since the 1930s, there has been state social welfare provision, and today charity is even celebrated through nationwide events and campaigns. Yet, what has not changed is the idea that the need of said charity—poverty—is predominantly the fault of the individual. Throughout ANZ’s colonial history, personal attributes rather than structural problems have been condemned. Implicit in this individualised approach is continued failure to acknowledge how processes of colonisation led to alienation and dispossession for some, and accumulation for others.

Poverty and hunger have been consistently justified by narratives of individualism and self-sufficiency and the myths of the level playing field, egalitarianism, and classlessness, which have in turn allowed a deserving/undeserving binary to flourish. There has been a persistent lack of trust afforded to those asking for assistance, leading to stricter procedures around obtaining support that further exacerbate recipients’ sense of social deficiency. Government policy inaction has then been supported by and solidified such narratives.

What is particularly striking is how in the nineteenth century, despite the severe lack of employment and multiple economic depressions, focus remained on the fecklessness of the poor, justifying a lack of social assistance. Fast forward to today and, although the method of food charity may have changed, and the breadlines replaced with appointment times, the victim-blaming mentality has prevailed, again justifying scant social assistance to the point that, just as soup kitchens were deemed unnecessary when they first appeared in the 1850s and seen as generating their own demand, foodbanks have been condemned as a creation *of* the poor rather than for them, castigating those in need rather than the need for food charity.

The genealogy reveals, therefore, that claims that neoliberal policies are primarily to blame for individualistic and victim-blaming attitudes is flawed: this stance has been present since the introduction of capitalism and a system based on scarcity and inequality; while foodbanks might be affiliated with neoliberal politics in particular, their ideological foundations were established beforehand. Blaming neoliberalism specifically moves the focus away not only from the detrimental impacts of capitalism in general but colonisation, of which widespread hunger and food charity in ANZ are products. Shifting the focus to the colonial as opposed to neoliberal roots of hunger and charity brings deeper issues around discrimination and inequality to the surface, highlighting how these have shaped institutional policies and practices and continue to dictate access to resources and power.

### Points of resistance and political action

Notably, food charity clientele has changed: while soup kitchens, in advance of a robust social welfare system, were primarily frequented by the unemployed during distinct periods of economic recession, foodbanks are primarily frequented by beneficiaries on a consistent basis, regardless of underlying economic conditions. This difference is then reflected in the main point of resistance for the need for food charity. For soup kitchens, it was strongest from those expected to use them, namely the unemployed, their struggle being founded on their right to work. Unemployment unions were particularly vocal throughout the nineteenth and twentieth centuries, and demand for work—and food—even erupted into riots in response to the Great Depression. Foodbank resistance, specifically in phase 1, has primarily originated from those running them and focused on the overzealous social welfare cuts made in the 1990s. Although the explanations for need remained individualised throughout, the response was collective across both periods.

The 1930s and 1990s also reflect two main moments in which human rights came into the limelight in ANZ: in the ‘30 s they were used by the government as a foundation for the social welfare system, and in the ‘90 s they were used by civil society and the Church as the foundation for political action. Markedly, both these periods not only brought national hunger and poverty to the public’s attention, but a more significant proportion of the public were personally having to contend with these issues.

With economic recovery, such topics were no longer deemed politically pressing. Today, the media lens is rarely on those who require food parcel assistance, apart from as pitiable beings: foodbanks users tend to “look more like objects of compassion than potential allies” (Poppendieck 1995, p. 27). This lack of comradery is reflected in the fact that there are more reports published than revolts planned; more “performance protests” than demonstrations of socially driven political anger at the continued existence of hunger. For instance, while in 1996 a rally was held in Ōtepoti-Dunedin’s central plaza in protest of the existence of foodbanks, today in that same space the public creates attractive displays of canned food to preserve them. Food charity recipients are both blamed for their situation and stripped of their political agency to organise and resist.

In addition, the corporate appropriation of food charity has assisted in simplifying the issue of hunger into a matter of “more food”, and supermarkets in particular have helped cement public donations as the extent of public engagement, decreasing the amount of meaningful action. Instead, the main point of resistance today could be thought of as the food rescue organisation with its “one stone, two birds” approach to food waste and hunger/food charity. The point of resistance has been further removed from those who are hungry and from those helping those who are hungry to those supplying those helping those who are hungry.

The coupling of foodbanks with the corporate need to address its food waste issue could be seen to have further entrenched food charity as a secondary food system (Tarasuk and Eakin 2005). Entrenchment of food charity became less insidious and more shameless when connections were made with food waste and, in conjunction with this, climate change, justifying the existence of foodbanks and foodbank subsidiaries, and seeming to transcend the “primary” reason for foodbanks, i.e., domestic hunger. Instead, hunger is being used to solve the issue of food waste (Arcuri 2019; Lohnes 2021).

The foodbank no longer merely symbolised injustices but a way in which to *address* injustices, rationalising them to the point that their disbandment is considered socially detrimental. This protection of unsustainable food production has not only marginalised the root causes of food poverty (Tikka 2019) but feeding people with food waste is recognised as an affront to human dignity and basic human rights (McIntyre et al 2017). Those approaching foodbanks must receive “what are essentially society’s leftovers” (Tarasuk and MacLean 1990, p. 332): “residual food for residual citizens” (Silvasti 2015, p. 478).

Meanwhile, the Church, for all its politically significant action, remains in a bind. Since the colonial days, there has been a clear sense of Christian duty to help those in need mixed with judgement as to who is and who is not worthy of such help. Contradictions have remained in regard to the intentions of church-run foodbanks: the provision of charity for all, but with conditions. Churches have also had to negotiate the increasingly contested space between the immediate need at the grassroots levels and the policies and practices causing this need. This has been achieved primarily through the ongoing publication of data and advocacy. The Salvation Army’s “State of the Nation” is a prime example. Despite the deeply rooted secularism in ANZ, this report published by a Christian organisation speaks to both the wider public and the state, reflecting the Church’s significant influence within the country’s social and political spaces.

This genealogy of hunger thereby accentuates how, although marketisation evidently depressed political action, the voices of those in need have never actually been heard, let alone been incorporated into effective solutions. Economic casualties of the Great Depression were even physically removed after the 1930’s riots. The difference today is that such suppression is more subtle: the hungry are self-disciplined, silencing themselves by the atomisation of shame. Focusing on neoliberalism’s muzzling of civil society, however, detracts from the fact that such disregard of community sentiment goes to a deeper level of governance and more submerged systemic implementations. The apparent convenience of food charity has always trumped public opinion of it.

### Individualisation of blame

That people are left to feel ashamed of their predicament leads to the third point of comparison: the silencing of victims through shame brought about by social perceptions of food charity dependency. Disapproval for soup kitchens was aimed more at the shame of such institutions being a symbol of social stratification. The colonial tenets of self-sufficiency and equal opportunities manifested through land availability placed the onus of poverty on those who were poor. However, because soup kitchens were an emblem of national and thereby collective shame, this qualified resistance to the wider societal narratives about the root causes of hunger.

Within the foodbank period as a whole, shame has been directed more at the food parcel recipient as opposed to the foodbank. This individualised shame has silenced people and organically caused them to socially exclude themselves: they do not take action (Hojman and Miranda 2018); they are politically castrated and even seemingly complicit within their plight in that their disempowerment is recast as laziness and demotivation (Cresswell Riol 2021). The lack of human rights talk has also assisted with the internalisation of blame: not recognising themselves as citizens with rights that should be respected by the state and obscuring who is accountable.

Therefore, a main point of difference between the first two periods and the third is the increased outrage of those who find themselves dependent being turned inwards: what should be anger at structural issues has morphed into self-blame. People do not feel that their grievances, and thereby any remedial action, are justified. This is in stark contrast to the objections of the unemployed in the nineteenth and twentieth centuries over soup kitchens, or the protests in the early 1990s over foodbanks.

Although shame at having to depend on food charity has been a constant across the periods, there is a difference in how it has been experienced: the collective sense of shame in the soup kitchen period led to widespread resistance, while internalisation of shame in the foodbank period by those dependent on food charity has led to social inertia. Much research on foodbanks focuses, instead, on the consistent sense of shame in having to use charity, thereby disregarding the solidarity spawned from the perception of collective shame. Acknowledging this distinction reinforces the importance of appreciating hunger as an issue of societal rather than individual shame.

## Wider implications

Firstly, hunger needs to be reframed as an emergency begot by systemic issues, and not hidden behind other social issues let alone individualised: what can be seen in ANZ’s response is an obscuring of the issue at best and normalisation at worst. In the current climate, hunger is being managed rather than eliminated, and not being granted the gravity it deserves in and of itself.

While soup kitchens were seen as a temporary necessity tied to specific economic downturns, today, hunger persists amidst booming economies. Being able to take a long gaze through this genealogy of hunger reveals that it is no longer regarded as a crisis—which implies exceptional and deplorable as well as temporary circumstances—but a new reality. This perpetuates the belief that hunger is not an issue for debate. Consequently, it is not measured and accounted for. This then renders it invisible, maintaining the myth that it is not an issue and that public and government inaction is subsequently justified.

On the other hand, with the transformation of blame and shame from society to individual foodbank users, there is no longer any need to deny the existence of charity. In fact, it is celebrated as evidence of wider society’s contribution to help those who refuse to help themselves. In this respect, the institutionalisation of foodbanks has made sense.

Although food charity was the default answer to hunger since colonisation, setting the standard despite protestations from various fronts, soup kitchens were acknowledged as a social anomaly, whereas today foodbanks are a social normality. Looking across the historical periods, it is evident that food philanthropy has been increasingly used to depoliticise hunger to the point that there is no longer any significant debate, no moral outrage, and no public protest. Complacency has abounded due to the cementing of the belief that hunger is being addressed by the voluntary and corporate sectors together with the recent push for waste diversion and climate change mitigation.

It is through its visibility that hunger can be appreciated as a fault of society not the individual, and this shift can revive a sense of collective shame, but at a human rather than nationalistic level. In order to transform how we address hunger, there clearly needs to be a paradigm shift in how we understand the role of charity, i.e., as a short-term crisis not a normal part of life, despite poverty and inequality being inherent parts of neoliberalism.

Secondly, focus should be placed on state inaction rather than economic system rationale: economic theory alone cannot explain hunger because it is not a result of anonymous market forces. Decisions are made by those with political power, and policies that lead to and exacerbate hunger are a result of social and political relations. In the context of ANZ’s historical response to hunger, there has been a state stance rooted in colonial and capitalist tenets that has endured. Even in the halcyon days of the social welfare state, not everyone benefited from the social welfare reforms, particularly Māori. Structural discrimination has curbed access to food, compounding the obstacles afforded by poverty.

Similarly, it is not about foodbank action and incapability. Charity itself is not at fault: the fault lies in the fact that people are having to rely on food charity in a high-income state due to government inaction. This was well-recognised from the soup kitchen period, which demonstrates how the persistent condemnation of food charity as an institution of shame has detracted from the wider social, political, and economic forces at play and the blame that should be placed on inert governance.

Thirdly, the voices of the food insecure should be given priority. It is apparent that, since the soup kitchen period, not only have their voices been silenced by the institutionalisation and normalisation of foodbanks but the recognition that they are “victims” of political transgressions has been nullified. Both aspects have been increasingly hidden behind campaigns and additional causes to the point that the supermarket is recognised more as a good corporate citizen than the food parcel recipient as a citizen at all.

Because soup kitchens were viewed negatively by society, churches were the predominant institutions willing to be associated with them. Through their normalisation, foodbanks are regarded much more positively, and we now have a broad range of public and private institutions tied to the foodbank brand for corporate social responsibility and goodwill beyond churches. There clearly needs to be a re-evaluation of food charity as an unfavourable solution to hunger, and collective action that gives rise to alternatives.

All three of these implications tie into the fourth: this genealogy reveals how hunger has consistently been recognised as an issue of charity rather than justice. Despite its opportunity for generosity, connection, and even solidarity, charity in whatever guise has always symbolised a lack of justice. Arguably, nowhere are the historical injustices that are at the core of social, political, and economic inequalities more evident than in the philanthropic sector.

Social welfare provision has remained rooted in charity because hunger has been consistently presented as a problem that is being addressed by charity, even though it is not doing so effectively. The bitter irony of this is that it has enabled the core historical reasons behind the present issues charity is addressing—the past realities that forced individuals and communities into such oppressive situations—to be ignored, perpetuating a lack of understanding and reflection.

## Conclusion

Through this overview of the historical context of ANZ, we have sought to demonstrate how a neoliberal understanding of food charity detrimentally impacts how we then address hunger. Foodbanks might be the most recent configuration but they are arguably an entrenched corporatised version of the nineteenth century soup kitchen, with the state and business today’s primary almsgivers (Booth 2014). Although it is important to recognise that neoliberal policies exacerbated hunger and economic inequality in the ‘80 s and ‘90 s and led to the institutionalisation of foodbanks—particularly through the marketisation and corporatisation of foodbanks and the amalgamation of food waste and food poverty—it should also be acknowledged that the country is built upon dominant colonial and capitalist ideologies that perpetuate and even uphold these social issues, shaping the policies and practices that have led to hunger, providing the technicalities required to excuse the administration of scant or no assistance, and repressing and disregarding civil society’s protestations. On the other hand, although the shame surrounding charity has been consistent, focus on how shame is now primarily carried by the individual has overshadowed that of the collective.

All three points of comparison—individualistic narratives, points of resistance and political action, and individualisation of blame—point to, firstly, the need to appreciate the deeper systematic nature and societal implications of hunger, and secondly, the role of food charity in addressing this issue beyond neoliberalisation. Charity has always taken up state responsibilities; always filled a social welfare vacuum; always been a replacement for justice. This signals that there needs to be an assessment of the social norms and laws that set the framework for philanthropy and the moral and political dimensions of charity as it sits within capitalism, and an aspiration to social conditions that render food charity in its various forms redundant.

## Footnotes

1 A division of the Ministry for Social Welfare responsible for managing benefit payments.
